# HKUST-1@IL-Li Solid-state Electrolyte with 3D Ionic Channels and Enhanced Fast Li^+^ Transport for Lithium Metal Batteries at High Temperature

**DOI:** 10.3390/nano11030736

**Published:** 2021-03-15

**Authors:** Man Li, Tao Chen, Seunghyun Song, Yang Li, Joonho Bae

**Affiliations:** Department of Nano-Physics, Gachon University, Seongnam-si, Gyeonggi-do 461-701, Korea; liman19921224@gmail.com (M.L.); chentao1191470261@gmail.com (T.C.); songsh13@naver.com (S.S.); liyang941019@gmail.com (Y.L.)

**Keywords:** composite solid electrolyte, 3D ionic nanochannel, high ionic transference number, solid-state lithium metal batteries, high temperature

## Abstract

The challenge of safety problems in lithium batteries caused by conventional electrolytes at high temperatures is addressed in this study. A novel solid electrolyte (HKUST-1@IL-Li) was fabricated by immobilizing ionic liquid ([EMIM][TFSI]) in the nanopores of a HKUST-1 metal–organic framework. 3D angstrom-level ionic channels of the metal–organic framework (MOF) host were used to restrict electrolyte anions and acted as “highways” for fast Li^+^ transport. In addition, lower interfacial resistance between HKUST-1@IL-Li and electrodes was achieved by a wetted contact through open tunnels at the atomic scale. Excellent high thermal stability up to 300 °C and electrochemical properties are observed, including ionic conductivities and Li^+^ transference numbers of 0.68 × 10^−4^ S·cm^−1^ and 0.46, respectively, at 25 °C, and 6.85 × 10^−4^ S·cm^−1^ and 0.68, respectively, at 100 °C. A stable Li metal plating/stripping process was observed at 100 °C, suggesting an effectively suppressed growth of Li dendrites. The as-fabricated LiFePO_4_/HKUST-1@IL-Li/Li solid-state battery exhibits remarkable performance at high temperature with an initial discharge capacity of 144 mAh·g^−1^ at 0.5 C and a high capacity retention of 92% after 100 cycles. Thus, the solid electrolyte in this study demonstrates promising applicability in lithium metal batteries with high performance under extreme thermal environmental conditions.

## 1. Introduction

Over the past two decades, Li secondary batteries have been recognized as the dominant energy storage devices, since the combination of their high energy density, light weight, and good long-term stability makes them highly attractive for portable and efficient electronic products [1,2]. However, the formation of dendritic Li during the plating/stripping process and safety issues caused by traditional liquid organic electrolytes such as leakage and fire are the major problems that make it difficult to meet the demands of high-power applications using lithium ion batteries [3,4]. In addition, there is a growing demand for batteries to survive and operate under extreme thermal and environmental conditions, for which such lithium batteries need to be optimized and developed urgently. The thermal effects on individual materials which make up a battery have been researched in many recent research works since temperature plays a dominant role in the charge storage mechanism in batteries in practical applications [5,6,7,8]. However, the major challenge of batteries operating at high temperatures is the failure of separator and electrolyte. A thin polymeric film is usually used as the separator, but it would soften and shrink when long-term exposed to high temperature, resulting in an electrical short circuit [9]. Liquid organic electrolytes in conventional batteries is easily inclined to thermal runaway under high-temperature conditions, because of low boiling points and high flammable properties [10]. To solve the above problems, solid-state electrolytes (SSEs) have been regarded as promising materials for electrical energy storage in batteries. 

Ionic liquid (IL) is regarded as a kind of green solvent with many excellent properties of negligible volatility, nonflammability and high ionic conductivity, and thermal stability [11]. As reported, ILs can keep stable under high temperatures, not producing significant amounts of volatile products until they reach ~300 °C [12]. However, the exploitations of the applications of ILs in lithium batteries are limited due to the lacking of thermal stable separators at high temperature. IL–polymer composite gels have been researched recently, but the rapid decay of their mechanical properties with temperature hinders the extension of the operating range [13].

Metal–organic frameworks (MOFs) are microporous crystalline materials featuring high surface areas and tunable, highly ordered pore structures, which make them promising candidates for designing energy storage materials [14,15,16]. Because of their highly tunable and ordered pore structures, MOFs are often introduced and used as hosts for designing electronic and ionic composite materials [17]. MOF-based proton-conducting electrolytes have received attention because of their loading of protonic inorganic and organic molecules, such as H_2_O, H_2_SO_4_, and imidazole [18]. Hybrid proton-conducting electrolytes of biomolecule/metal–organic framework (DNA@ZIF-8) membranes were reported by Guo et al., exhibiting a high proton conductivity of 3.40 × 10^−4^ S·cm^−1^ at 25 °C [19]. For ionic conductive electrolytes, a class of metal-ion-loaded MOFs (MIT-20-X (X = Li, Na, Mg)) functioning as single-ion solid electrolytes was reported [20]. Certain stoichiometric anions were applied to bind to Cu-based MOF to fabricate Mg^2+^-, Li^+^-, and Na^+^-loaded electrolyte materials, which exhibited ionic conductivities of 8.8 × 10^−7^, 4.4 × 10^−5^, and 1.8 × 10^−5^ S·cm^−1^ and activation energy values of 0.37, 0.29, and 0.39 eV at 25 °C. Additionally, MOFs used in designing SSEs can expand the operating range of batteries to function capably and safely in extreme conditions, attributed to their thermal and mechanical stability [21]. A composite solid electrolyte was made by combining MOF with ionic conductive polymer (PEGDA) and was assembled in Li/LiFePO_4_ cells, exhibiting good cycle performance at 60 °C with an average discharge specific capacity of 151 mAh·g^−1^ at 0.5C [22]. Unfortunately, when using the above MOF–polymer electrolyte, the slow recrystallization kinetics of the ubiquitous polymers results in a narrow operating temperature range (28–60 °C). 

**Figure 1:**
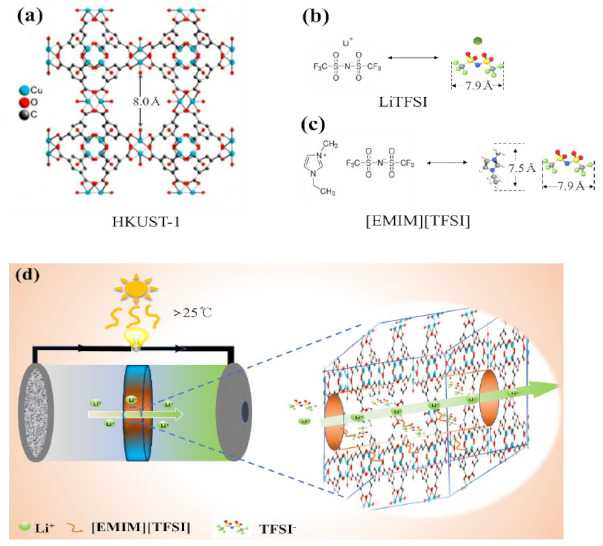
Molecular structures of (**a**) HKUST-1 metal–organic framework (MOF), (**b**) LiTFSI salt, and (**c**) [EMIM][TFSI] ionic liquid, and (**d**) schematic illustration of Li^+^ transport in 3D ionic nanochannels of solid electrolyte.

Hence, we propose a solid-state electrolyte obtained using the 3D ionic channels of MOF to immobilize a Li salt containing [EMIM][TFSI] ionic liquid (IL-Li salt). HKUST-1(Cu_3_(BTC)_2_) MOF modified liquid electrolyte has been reported to control the ion transport in LiTFSI liquid electrolytes and enable a stable Li-metal anode with retarded Li dendritic growth, because the pore diameter of HKUST-1 is about 8Å and slightly larger than the lengths of the TFSI anion [23]. In this work, considering the size of the TFSI anion (7.9 Å) and EMIM cation (7.5 Å) in ionic liquid and Li salt as shown in Figure 1a–c, the well-known HKUST-1 was also chosen as the appropriate host candidate among thousands of reported MOF species [24]. The angstrom-scale pores of HKUST-1 enable restrict the TFSI and EMIM ions’ transport to realize stable Li electrodeposition in a practical large temperature range [25]. The restriction of TFSI anions could provide a weaken coordination environment and improve mobility of Li^+^ ions, and IL-Li salt would maintain its dynamic mobility with high ionic conductivity when encapsulated in the channels of MOF as shown in Figure 1d. In addition, HKUST-1@IL-Li with 3D nanochannels possessed abundant direct contact points for the electrode with Li ions inside; thus, the interface between solid electrolyte and electrode was actually wetted with the guest ions at the atomic scale to turn the primitive solid–solid contact into “nanowetted” interfaces to decrease the interfacial resistance and ensure a stable Li metal plating/stripping while suppressing Li dendrites. This solid electrolyte, named as HKUST-1@IL-Li, with enhanced interfacial contact for fast Li^+^ transport and high thermal stability enable promote the fabrication of Li metal batteries possessing stable cycle performance at high temperatures. However, to our best knowledge, there have been no reports on the high-temperature performance of HKUST-1@IL-Li solid electrolyte used in lithium metal batteries thus far. 

## 2. Experimental Section

Synthesis of HKUST-1 MOF: The solvothermal synthesis method was used to prepare MOF of HKUST-1. Two solutions mixed with *N*-*N*-dimethylformamide (DMF, Sigma-Aldrich Inc., Billerica, MA, USA), distilled water, and ethanol with a volume ratio of 1:1:1 were prepared. 0.966 g, 4 mmol Copper (II) nitrate trihydrate (Cu(NO_3_)_2_·3H_2_O, DAE JUNG Inc., Gyeonggi-do, Korea) and 0.42 g, 2 mmol 1,3,5-benzenetricarboxylic acid (H_3_BTC, Sigma-Aldrich Inc., Billerica, MA, USA) were dissolved in each solution, separately. The two solutions were mixed homogeneously, then the mixture was transferred into 50 mL Teflon-lined stainless steel autoclave and reacted at 85 °C for 24 h. The product was washed three times with DMF followed by washing with dichloromethane for three days, during which the dichloromethane (SAMCHUN Co., Seoul, Korea) solvent was pour out and freshly replenished twice daily. The sample was then vacuum-dried at 80 °C overnight. 

Preparation of HKUST-1@IL-Li ionic conductor: IL–Li (1 M) was obtained by dissolving LiTFSI (Sigma-Aldrich) in [EMIM][TFSI] ionic liquid (Sigma-Aldrich Inc., Billerica, MA, USA). Next, 80 mg of activated HKUST-1 was soaked in the 1 M LiTFSI in [EMIM][TFSI] and stirred for 24 h. After the IL–Li filled the porous MOF and was solid, the remaining solvent was filtered out and the ionic conductor was heated at 85 °C under vacuum overnight. Then, the HKUST-1@IL-Li powder was pressed into pellets under 8 T of force for the electrochemical testing. 

Characterization: Powder X-ray diffraction (XRD)data were recorded using an advanced diffractometer with Cu Kα (λ = 1.541 Å) in a 2θ range of 5°–30° with a scanning rate of 5° min^−1^. Scanning electron microscopy (SEM) and energy dispersive spectrometry (EDS) were performed using a scanning electron microscope (S-3400N, Hitachi, Japan) to observe the morphologies of the products and determine the chemical composition. N_2_ adsorption-desorption isotherms were recorded on an adsorption analyzer (TriStar 3000V 6.05 A, Micromeritics, UK). TGA was performed with a 5 °C min^−1^ scanning speed in N_2_ atmosphere on an SDT Q600 system. Electrochemical impedance spectroscopy (EIS, 0.1–1 MHz) and Li plating-stripping cycles were collected with an electrochemical workstation (VersaSTAT 4, Princeton Applied Research, DE, USA). The potentiostatic polarization measurement and battery charge–discharge performance was obtained with an automatic battery cycler (WBCS3000, Wonatech, Korea).

The ionic conductivity (σ, S·cm^−1^) was measured by sandwiching the pellets material between two stainless steel plates in coin cells. Then, ionic conductivity was determined based on

σ = L/ (R × S)(1)

R is the end point of the semicircle and used as the ionic resistivity, Ohm; L is the thickness of pellet, cm; and S is the area of the pellet, cm^2^.

Lithium symmetric cells were assembled for the measurements of the Li ion transference number (t_Li+_) and Li stripping/plating process. The HKUST-1@IL-Li electrolytes were sandwiched between two Li-foil electrodes to assemble Li/ HKUST-1@IL-Li /Li symmetric cells. t_Li+_ was measured by combining AC impedance and potentiostatic polarization measurements. A constant DC voltage (ΔV, 50 mV) was applied to the cell, then the current would decease until achieving a steady state. The initial current (I_0_) and the steady-state current (I_s_) were obtained during the process. An AC impedance test (1–10^6^ Hz) was performed to obtain the initial bulk resistance ($R_{b}^{0}$(before), $R_{b}^{s}$(after)) and the steady-state interfacial resistance ($R_{1}^{0}$(before), $R_{1}^{s}$(after)) before and after polarization measurement. t_Li+_ was then calculated by using the equation:

(2) $$t_{Li+}=I_{s}\;R_{b}^{0}\left[\Delta V-I_{0}\;R_{1}^{0}\right]/I_{0}\;R_{b}^{s}\left[\Delta V-I_{s}\;R_{1}^{s}\right]$$

Li stripping/plating processes were carried out using Li/ HKUST-1@IL-Li /Li cells by galvanostatic charging–discharging method at a current density of 0.5 mA·cm^−2^.

Battery assembly: A cathode slurry of commercial LiFePO_4_, HKUST-1@IL-Li, and carbon black (Super-P) in a weight ratio of 5:5:2 was well mixed using N-methyl-2-pyrrolidone (NMP) as a solvent. Then, the slurry was uniformly coated on an aluminum current collector and cut into cathode plates after drying at 80 °C overnight under a vacuum. To minimize interfacial resistance, 80 mg of HKUST-1@IL-Li was pressed onto the cathode mixture under 8T (24 MPa) of force to make a bilayer pellet, to be used as a cathode and separator in SSBs. The CR2032 type cells were assembled in a glove box using Li foil as the anode and the bilayer pellet as the cathode and solid electrolyte. Adding about 6 μL of electrolyte was done to exclude possible gas, and to ensure a good contact between electrolyte pellet and Li metal anode. The specific capacity was calculated based on the active materials in the cathode, which corresponded to an area loading of ~0.75 mg·cm^−2^. The cycling performance were measured with an electrochemical window of 2.5–4.0 V at different C rates (0.5, 0.8, 1, and 2C) for five cycles each and followed by long-cycle life performance tests at 0.5C at room temperature and 100 °C. The charge–discharge rate of 1C used here is 170 mA·g^−1^.

## 3. Results and Discussion

**Figure 2:**
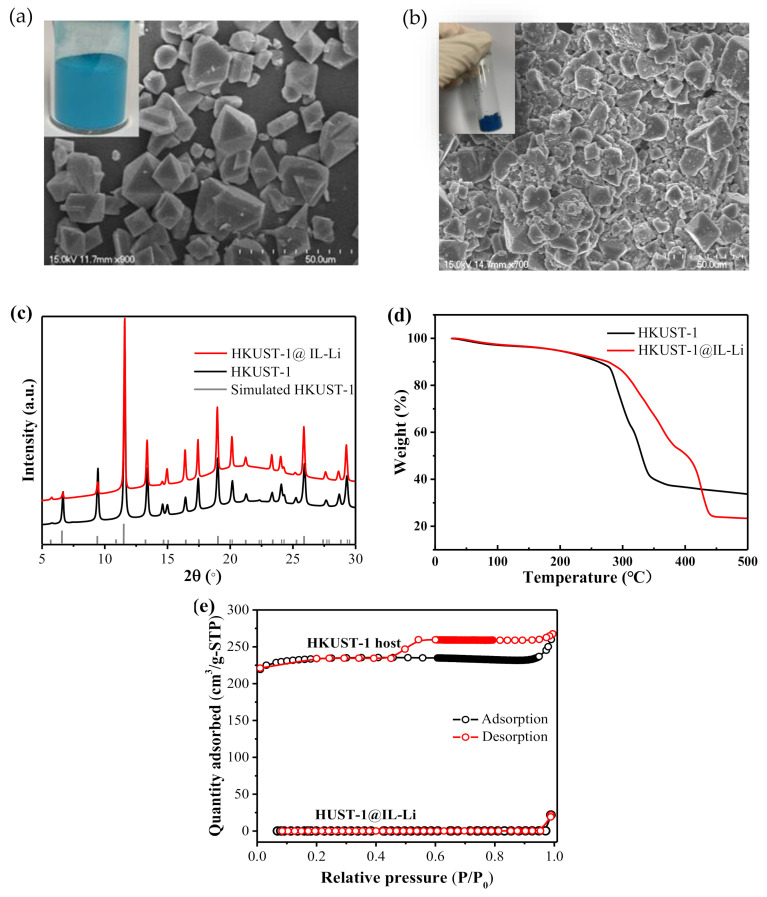
Structure and thermal characterizations of HKUST-1@IL-Li electrolytes. Scanning electron microscopy (SEM) images of (**a**) HKUST-1 particles and (**b**) HKUST-1@IL-Li electrolyte (insets: photographs of pristine HKUST-1 and HKUST-1@IL-Li). (**c**) X-ray diffraction (XRD)patterns of simulated HKUST-1, pristine HKUST-1, and HKUST-1@IL-Li. (**d**) Thermogravimetric analysis patterns and (**e**) Nitrogen adsorption/desorption isotherms of MOF host and HKUST-1@IL-Li.

HKUST-1 was synthesized using the solvothermal method. SEM image in Figure 2a shows octahedral particles of pristine HKUST-1 with an average size about 15 μm. A similar morphology of HKUST-1@IL-Li was observed in Figure 2b, indicating the chemical stability of the MOF against IL-Li salt. XRD pattern of pristine HKUST-1 in the 2θ range of 5–30° displayed typical diffraction peaks at θ = 6.75° and 11.60° in Figure 2c, suggesting that as-synthesized materials were MOF of HKUST-1 [26]. Identical reflection peaks between HKUST-1@IL-Li and pristine HKUST-1 in XRD patterns demonstrate that a structure intactness of the MOF host was maintained after being filled with IL-Li salt, also proven by SEM morphology of HKUST-1@IL-Li (Figure 2b). The intensity dropping observed in the first reflection peak might have resulted from the disordered IL–Li salt guest ions, as previously reported [26]. Because the thermal stability of an electrolyte played an important role in the safety of the battery, thermogravimetric analysis (TGA) was applied in N_2_ to test the thermal property of the HKUST-1@IL-Li ionic conductor. The thermal stability was not affected after filling the pores of the MOF host with the ionic liquid, as the mass loss tendency of HKUST-1@IL-Li was similar to that of pristine HKUST-1 at <250 °C (Figure 2d). The samples exhibited a mass loss initially up to a temperature of 250 °C owing to the dehydration of the material, the weight change corresponding to approximately 3 mol of H_2_O per Cu_3_(BTC)_2_ [27]. A second weight change from the decomposition of the network was approximately 300 °C. Thus, the HKUST-1@IL-Li electrolyte enabled the promise of a wide operating temperature range for SSBs since it exhibited favorable thermal stability. HKUST-1 exhibited a microporous crystalline structure with a specific surface area of 950 cm^2^·g^−1^ and pore volume of 0.4 cm^3^·g^−1^. The surface area and pore volume of HKUST-1@IL-Li decreased to nearly zero, as shown in Figure 2e, suggesting successful encapsulation of IL-Li gest into the pore of the MOF host, which also proved by the SEM image with corresponding energy dispersive spectrometer (EDS) elemental mappings of HKUST-1@IL-Li in Figure S1.

**Figure 3:**
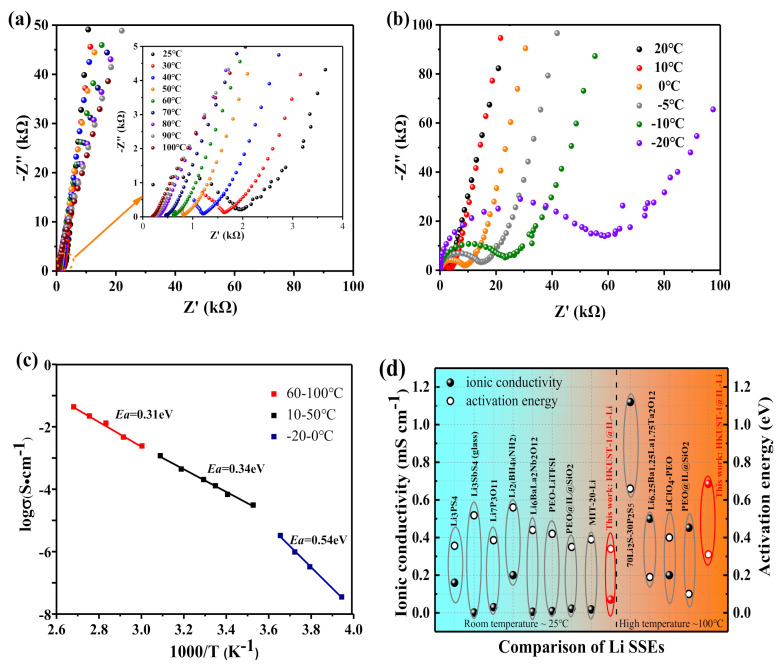
Lithium ion conductivity of HKUST-1@IL-Li ionic conductors: Nyquist plots of at different temperatures of (**a**) 25–100 °C and (**b**) −20–20 °C. (**c**) Arrhenius plots and corresponding *Ea* values of electrolyte under different temperature ranges. (**d**) Ionic conductivities and activation energies of HKUST-1@IL-Li electrolyte at room and high temperature in comparison with representative electrolytes: sulfides electrolytes Li_3_PS_4_ [28], Li_3_SbS_4_ (glass) [29], and 70Li_2_S-30P_2_S_5_ [30]; oxide electrolyte Li_7_P_3_O_11_ [31]; complex hydrides electrolyte Li_2_(BH_4_)(NH_2_) [32]; ceramic electrolytes Li_6_BaLa_2_Nb_2_O_12_ [33]; and Li_6.25_Ba_1.25_La_1.75_Ta_2_O_12_ [34]; polymeric electrolytes PEO-LiTFSI [35], LiClO_4_-PEO [36], and PEO@IL@SiO_2_ [35], and MOF based electrolyte: MIT-2-Li [20].

The ionic conductivities of the HKUST-1 and HKUST-1@IL-Li composite pellets were studied by alternating current (AC) impedance measurements at room temperature (25 °C). A significant reduction in resistance was observed after filling the HKUST-1 pores with the IL of [EMIM][TFSI] (Figure S3, Supporting Information). Moreover, from examining the ionic conductivities of the HKUST-1@IL-Li composites at different temperatures (<25 °C and >25 °C), the ionic conductivity increased with increasing temperature (Figure S4, Supporting Information). And the electrochemical impedance spectroscopy (EIS) of HKUST-1@IL-Li in the ranges of 25–100 °C and −20–20 °C are displayed in Figure 3a,b, respectively. The calculated ionic conductivities are 0.68 × 10^−4^ S·cm^−1^ at 25 °C, and 6.85 × 10^−4^ S·cm^−1^ at 100 °C (Table S1, Supporting Information) based on the measurements, which is sufficient for batteries in practical applications [20]. Furthermore, typical Arrhenius-liked temperature-dependent conductivities of ionic conductor were observed in Figure 3c, in that the conductivities increased linearly with increasing temperature.

**Figure 4:**
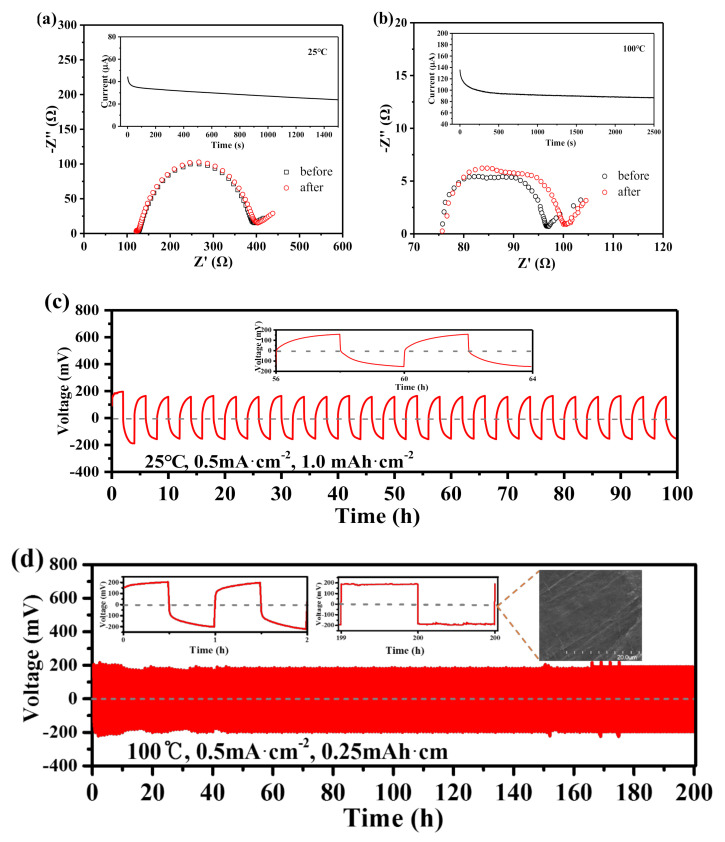
(**a**,**b**) EIS of Li|HKUST-1@IL-Li|Li cell at initial and steady states (insets: current–time profiles during polarization process) at room temperature, 25 °C and high temperature, 100 °C. (**c**,**d**) Galvanostatic cycling of Li plating/stripping at room temperature, 25 °C, with 0.5 mA·cm^−2^ current density, 1.0 mAh cm^−2^ areal capacity, and insets of the enlarged voltage profiles for 56–64 h, high temperature, 100 °C, with 0.5 mA·cm^−2^ current density, 0.25 mAh cm^−2^ areal capacity, and insets of the enlarged voltage profiles for 0–2 h, 199–200 h, and the surface SEM images of Li metal anode after the plating/stripping process.

The activation energies (*Ea*) calculated by using an Arrhenius equation in the ranges of 10–50 °C (0.34 eV) and 60–100 °C (0.31 eV) are significantly higher than those in −20–0 °C (0.54 eV). This is because activation energy was influenced by the thermally activated ion transport process under different temperature ranges [37]. The viscosity of IL in MOF pores increases and will even be frozen at −12°C [38], resulting in slow ion mobility and difficult thermal activation at low temperatures. By contrast, Li^+^ ions could transfer much more quickly in the ionic channels at high temperatures, which was also confirmed by the results of Li^+^ transference number (t_Li+_) measurements in Figure 4b. The ionic conductivities and *Ea* values for this HKUST-1@IL-Li electrolyte are in the same temperature ranges compared to those reported for solid electrolytes and shown in Figure 3d. These include the ceramic electrolytes Li_6_BaLa_2_Nb_2_O_12_ (0.006 × 10^−4^ S·cm^−1^, 0.44 eV at 25 °C) [33] and Li_6.25_Ba_1.25_La_1.75_Ta_2_O_12_ (0.5 × 10^−4^ S·cm^−1^, 0.19 eV at 100 °C),^34^ and the polymeric electrolyte PEO@IL@SiO_2_ (0.22 × 10^−4^ S·cm^−1^, 0.35 eV at 25 °C; 0.45 × 10^−4^ S·cm^−1^, 0.1 eV at 100 °C) [31]. Compared with these reported solid electrolytes, our HKUST-1@IL-Li materials have good ionic conductivities and lower activation energies both at room and high temperatures.

A high transference number intercalated electrolyte and Li anode could obviously suppress Li dendrites [39]. However, traditional batteries using liquid electrolytes (1 M LiTFSI in 1:1 (v/v) DOL/DME) have a limited Li^+^ transference number (t_Li+_) range of 0.2–0.4 at room temperature [40], because of the mobility of anions in electrolytes. High polarization and electrolyte decomposition also appear to decline battery performance when the anions accumulate at the anode electrode [41,42,43]. Hence, the immobility of anions in electrolytes is critically important for battery performance. In this work, the Li^+^ transference number (t_Li+_) was evaluated using a symmetric Li|MOF @IL|Li cell by the potentiostatic polarization method [44]. A high t_Li+_ value of 0.46 at room temperature (Figure 4a) was obtained compared to that of traditional liquid electrolyte at room temperature, which is attributed to the restricted transport of TFSI^−^ anions in the channels of HKUST-1, leaving a three-dimensional pathway for free and charge-balancing Li ion transport. Besides, it also showed a significant enhancement of the lithium ion transference number compared to that of other reported work using MOF as host for fabricating MOF/IL solid-state electrolytes due to the appropriate diameter of pores. For instance, Wang’s team got a Li^+^ transference number of 0.36 by using MOF-525 as host with an aperture size of about 12 × 7 Å [45]. The t_Li+_ of garnet Li_7_La_3_Zr_2_O_12_ (LLZO) SSE also dropped to 0.31, 0.21, 0.18, and 0.15 upon mixing with 5 wt %, 10 wt %, 20 wt %, and 30 wt % of UIO-67 MOF/IL SSE, respectively, as shown in previously reported work [46]. The temperature effect on the Li^+^ transport process was also investigated for Li|HKUST-1@IL-Li|Li symmetric cells, and a much higher t_Li+_ value of 0.62 (Figure 4b) at high temperature (100 °C) was obtained. It is very close to the DFT-MD (density functional theory based molecular dynamics) simulation of the HKUST-1-modified electrolyte in previous reported work, that the t_Li+_ was determined to be ~0.7 [23]. At high temperatures, lower IL viscosity has positive effects on ion mobility and electrode wettability, and increases the rate performance [47]. However, the resistance of the passivation layer formed on the surface of Li electrodes would decrease rapidly; the resulting increased ionic mobility realized a quick steady state in the symmetric cells at high temperatures [48]. In summary, compared with previously reported MOF based solid electrolytes [45,46], our electrolyte material had slightly lower ionic conductivity values but enhanced fast Li^+^ transport for lithium metal batteries because of the appropriate diameter of pores of HKUST-1 MOF host chosen in our work.

The primary failure modes of lithium metal batteries are caused by short circuiting due to the growth of dendrite-Li during the Li plating/stripping process [49]. Owing to the high interfacial resistance and reactivity between SSEs and lithium metal anodes, their compatibility is poor because of the loose contact with micro-gaps [35,50]. In this work, the HKUST-1@IL-Li solid electrolyte exhibited enhanced interfacial contact with the electrode, because of the crystal structure of the MOF scaffold with 3D ionic channels, which can not only restrict electrolyte anions and provide pathways to facilitate homogeneous Li^+^, but can also enable the IL–Li ions within to make contact with the surface of electrodes directly through open tunnels promoting a wetted interface between HKUST-1@IL-Li and electrode. Thus, the Li dendrites were effectively suppressed and ensure stable Li metal plating/stripping. The HKUST-1@IL-Li solid electrolyte was implemented in a 2032-type symmetric Li||Li cell for the test of Li plating/stripping at 25 °C and 100 °C. First, the HKUST-1@IL-Li electrolyte in the symmetric cell was subjected to an areal capacity of 1.0 mAh·cm^−2^ in 2 h segments and a high current density of 0.5 mA·cm^−2^ at room temperature, where it exhibited a stable overpotential of approximately 180 mV with good cyclability without short circuits within a long cycle time of 100 h during the process of Li electrodeposition in Figure 4c, suggesting the growth of Li dendrite was effectively suppressed. Then, the temperature was increased to 100 °C to examine the stability of the HKUST-1@IL-Li electrolyte at practically high temperature (Figure 4d). The slight fluctuations of initial overpotential was observed because of the activation process. Its overpotential approached approximately 200 mV at the 50th hour, then remained almost constant during the long subsequent Li electrodeposition time up to 200 h. A flat and smooth surface of Li metal anode was observed after plating/stripping process from the SEM image in Figure 4d, suggesting there is no obvious growth of vertical dendrites. The good cyclability in both room temperature and high temperature is attributed to significantly improved contact with Li metal anode and effectively suppressed growth of Li dendrite during contiguous Li electrodeposition. 

**Figure 5:**
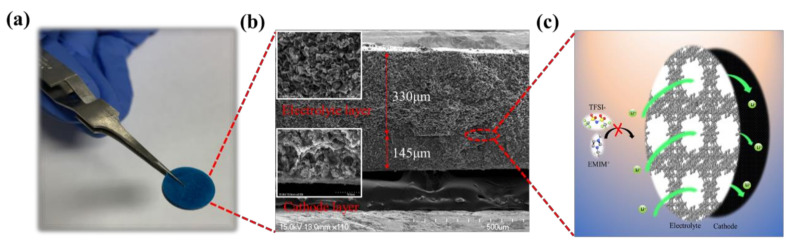

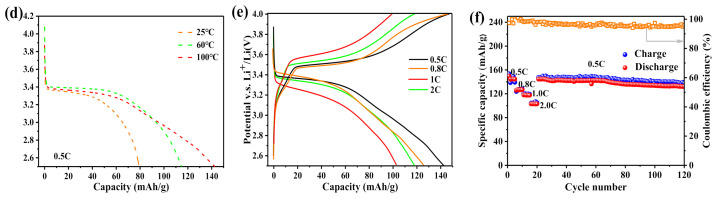
(**a**) Photograph and (**b**) cross-sectional SEM image of HKUST-1@IL-Li electrolyte/cathode bilayer pellet, and (**c**) schematic diagram of ion transport at the electrolyte–electrode interface. Electrochemical performance of a solid Li metal battery: (**d**) initial discharge curves at 25, 60 and 100 °C; (**e**) galvanostatic charge–discharge plots from 0.5 C–2.0 C; and (**f**) cycle life performance at 0.5 C for the LiFePO_4_|HKUST-1@IL-Li|Li cell at 100 °C.

To further demonstrate the practical applications of our solid electrolyte at high temperature, as-fabricated HKUST-1@IL-Li pellets were used as both the electrolyte and separator with LiFePO_4_ cathodes and Li anodes to assemble LiFePO4|HKUST-1@IL-Li|Li solid batteries. The electrolyte–cathode structure was observed through SEM images in a cross-sectional view (Figure 5a,b). The electrolyte–electrode interface was continuous, demonstrating that the HKUST-1@IL-Li (~330 μm thickness) electrolyte layer was having good contact with the LiFePO_4_ electrode (~145 μm thickness). From the magnified SEM image of the solid electrolyte layer, we can see the structure of the HKUST-1@IL-Li ionic conductor retained a similar morphology after being pressed, which ensured the rapid diffusion of Li ions. This porous architecture of the MOF host and good interfacial adhesion attributed lowered contact resistance and enhanced electrochemical performance during the charging and discharging processes.

The LiFePO_4_|HKUST-1@IL-Li|Li battery was evaluated in the temperature range of 25–100 °C at 0.5 C (1.0 C = 170·mA·g^−1^). The initial discharge curves (Figure 5d) show that the capacity increased with increasing temperature. The Li/LiFePO_4_ cell was then evaluated at rates ranging from 0.5 to 2 C at 100 °C, as shown in Figure 5e, in which the cell with the HKUST-1@IL-Li electrolyte exhibited a specific capacity of 144 mAh·g^−1^ at 0.5 C, while those at 0.8 C, 1 C, and 2 C demonstrated capacities of 125, 115, and 105 mAh·g^−1^, respectively. Further, we carried out extended cycling at both room temperature (Figure S5, Supporting Information) and high temperature (100 °C) (Figure 5f) conditions at 0.5 C and observed that 99% and 92% of the initial capacity were retained after 100 cycles, respectively. The good cycle life of this battery was attributed to the abundant 3D nanochannels of the HKUST-1@IL-Li electrolyte, which could offer wetted interfaces between the solid particles of the electrolyte and electrodes to boost overall Li^+^ transport. The efficiencies of over 92% observed under such extreme conditions demonstrated the high stability of the HKUST-1@IL-Li electrolyte when long-term exposed to extremely thermal environments while participating in electrochemical reaction processes. 

## 4. Conclusions

We present herein a simple but highly effective strategy for designing a solid-state electrolyte of HKUST-1@IL-Li by loading ionic liquid [EMIM][TFSI] in the pores of a microporous MOF host. Homogeneous Li electrodeposition was achieved to realize a good performance of a solid Li metal battery at extremely thermal environments. The ionic conductivity of the HKUST-1@IL-Li electrolyte could reach up to 0.69 × 10^−4^ S·cm^−1^ at 25 °C and 6.85 × 10^−4^ S·cm^−1^ at 100 °C. Furthermore, the angstrom-scale pores in the MOF of HKUST-1 can restrict TFSI^−^ anion transport, while providing a three-dimensional pathway for efficient Li^+^ transport, thus realizing a stable Li-metal anode with suppressed growth of Li dendritic and obtaining a high Li^+^ transference number, t_Li+_ (0.46 at 25 °C, 0.62 at 100 °C). As-assembled LiFePO_4_|HKUST-1@IL-Li|Li solid cells exhibit initial capacities of 144, 114, and 80 mAh·g^−1^ at 100, 60 and 25 °C, respectively. 92% of the initial capacity can be retained after 100 cycles at 0.5 C at a high temperature of 100 °C, indicating that the battery using HKUST-1@IL-Li as the electrolyte can function stably under extreme conditions. Therefore, this work provides a new strategy for designing a solid-state electrolyte with more efficient Li^+^ transport and good interfacial adhesion for a broad range of functional applications of Li metal batteries under harsh environments. 

## Data Availability

The data presented in this study are available in this article.

## Supplementary Materials

The following are available online at https://www.mdpi.com/2079-4991/11/3/736/s1, Figure S1: Surface morphology of electrolytes. SEM image of the HKUST-1@IL-Lielectrolyte powder and the corresponding element maps, Figure S2: Cross-sectional morphology of the cathode layer. SEM images of the cathode layer and the corresponding element maps, Figure S3: Nyquist plots of HKUST-1 and HKUST-1@IL-Li electrolyte at room temperature, Figure S4: Ionic conductivity of HKUST-1@IL-Li electrolyte in a wide temperature range, Figure S5: Electrochemical performance of a solid Li metal battery at 25 °C. (a) Typical charge–discharge voltage profiles from 0.1–2 C, (b) galvanostatic charge–discharge plots from 0.1–2.0 C, and (c) cycle life performance at 0.5 C of a LiFePO4|HKUST-1@IL-Li|Li cell, Table S1: Measured values for the parameters in Equation (1) and the corresponding calculated ionic conductivity values of HKUST-1@IL-Li electrolyte at different temperatures.
