# Supplementary material for: HKUST-1@IL-Li Solid-state Electrolyte with 3D Ionic Channels and Enhanced Fast Li+ Transport for Lithium Metal Batteries at High Temperature
Source: Nanomaterials (Basel). 2021 Mar 15;11(3):736. doi: 10.3390/nano11030736 (PMC7999087; doi:10.3390/nano11030736)
Supplement: Supplementary file 1 [file nanomaterials-11-00736-s001.pdf]

Supporting Information

HKUST-1@IL-Li Solid-state Electrolyte with 3D Ionic Channels and Enhanced Fast Li^+ Transport for Lithium Metal Batteries at High Temperature

Man Li [1] , Tao Chen [1], Seunghyun Song [1], Yang Li [1] *and* Joonho Bae [1,*].

[1]Department of Nano-physics, Gachon University, Seongnam-si, Gyeonggi-do, 461-701, Korea; liman19921224@gmail.com (M.L.); chentao1191470261@gmail.com (T.C.); songsh13@naver.com (S.H.S.); liyang941019@gmail.com (Y.L.)

* Correspondence: baejh2k@gachon.ac.kr

Number of pages: 6 (S1 to S6)

Number of tables: 1 (table S1)

Number of figures: 5 (figure S1 to S5)

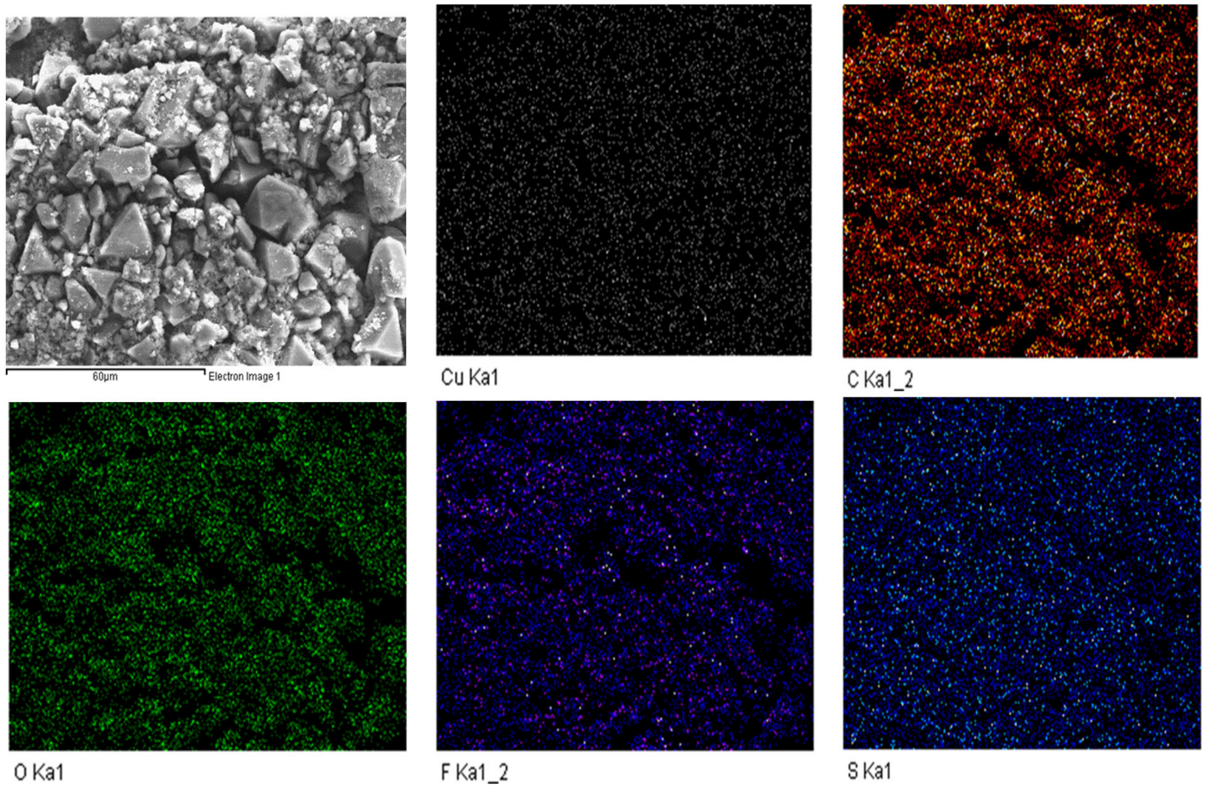

Figure S1. Surface morphology of electrolytes. SEM image of the HKUST-1@IL-Lielectrolyte powder and the corresponding element maps.

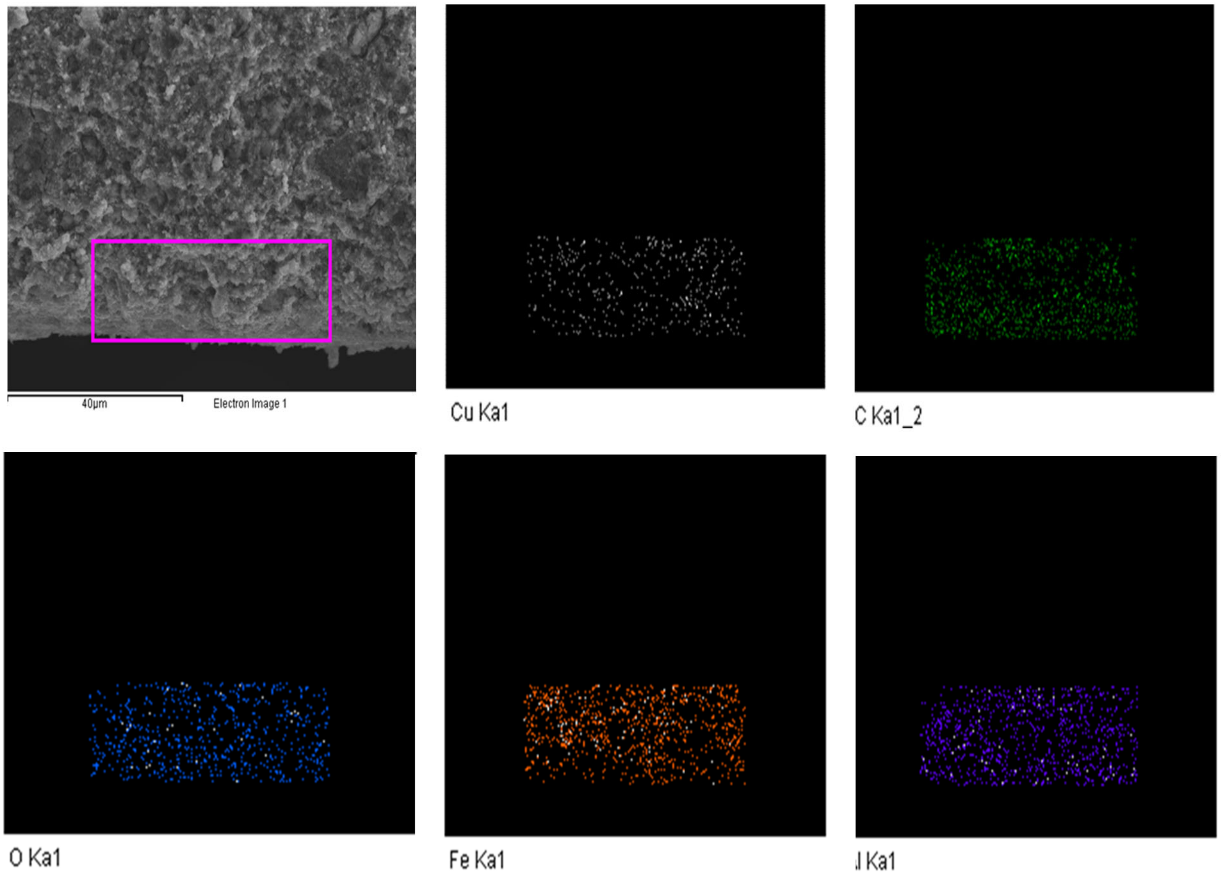

Figure S2. Cross-sectional morphology of the cathode layer. SEM images of the cathode layer and the corresponding element maps.

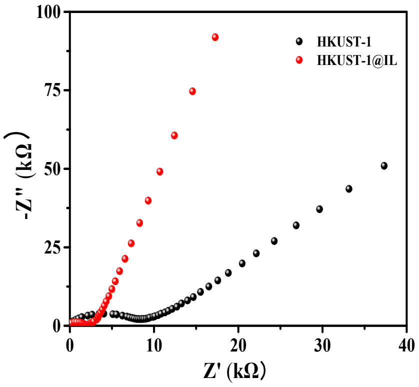

Figure S3. Nyquist plots of HKUST-1 and HKUST-1@IL-Li electrolyte at room temperature.

Table S1. Measured values for the parameters in Eq. (1) and the corresponding calculated ionic conductivity values of HKUST-1@IL-Li electrolyte at different temperatures.

>25 °C			< 25 °C		
T (°C)[a)]	R (Ω)[b)]	σ ($\times 10^{-4}$ S·cm^{-1})[c)]	T (°C)	R (Ω)	σ ($\times 10^{-4}$ S·cm^{-1})
25	1975	0.687	20	2511	0.540
30	1642	0.826	10	3478	0.390
40	1212	1.119	0	8852	0.153
50	816	1.662	-5	14812	0.092
60	617	2.198	-10	23244	0.058
70	469	2.892	-20	58752	0.023
80	314	4.320			
90	257	5.278			
100	198	6.851			

a) T: temperature; b) R: resistance; c) σ: ionic conductivity.

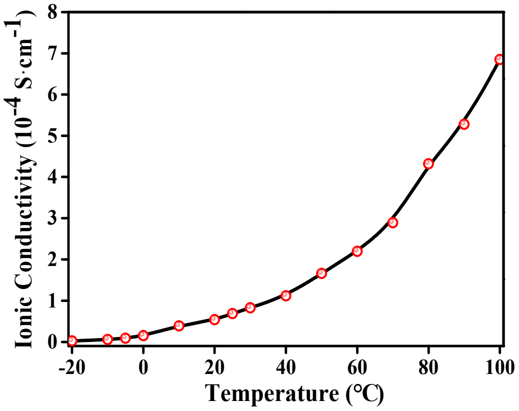

Figure S4. Ionic conductivity of HKUST-1@IL-Li electrolyte in a wide temperature range.

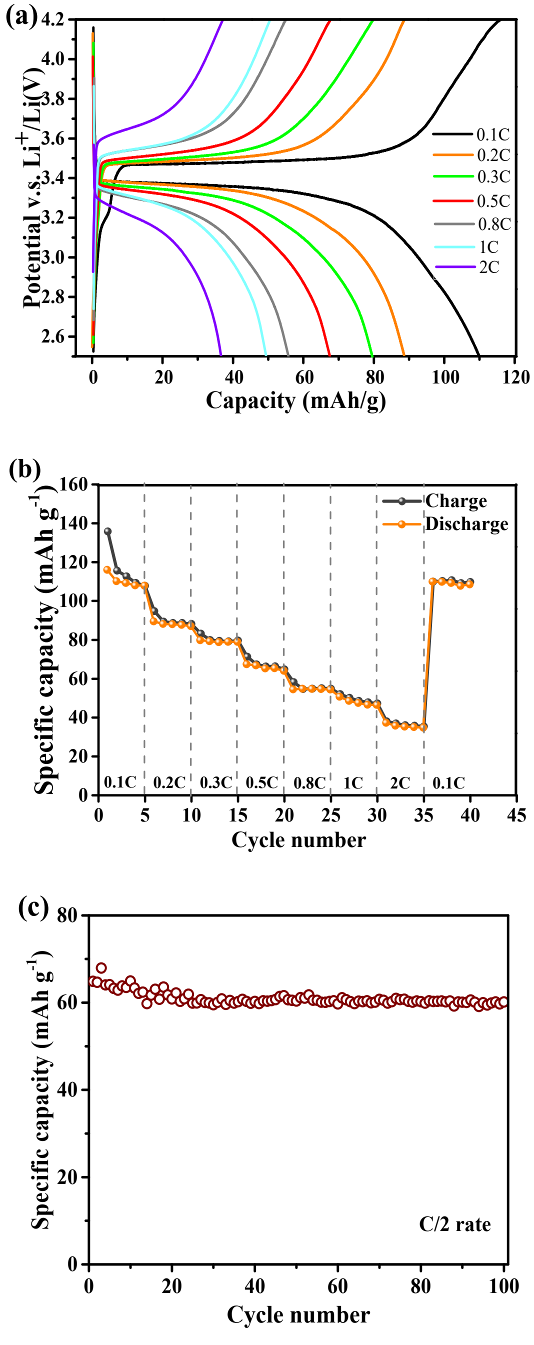

Figure S5. Electrochemical performance of a solid Li metal battery at 25 °C. a) Typical charge–discharge voltage profiles from 0.1–2C, b) galvanostatic charge–discharge plots from 0.1C–2.0C, and c) cycle life performance at 0.5C of a $LiFePO_4$|HKUST-1@IL-Li|Li cell.
